# Customized batch fabrication of highly sensitive thin capacitive soft sensors based on high dielectric constant composite polymers

**DOI:** 10.1038/s41598-025-27387-x

**Published:** 2026-01-12

**Authors:** Ali Ghanbari, Ahmet Arif Colakoglu, Ahmad Serjouei, Samit Chakrabarty, Ronaldo M. Ichiyama, Peter R. Culmer, Ali Alazmani

**Affiliations:** 1 School of Engineering, University of Lancashire, Preston, PR1 2HE UK; 2 Department of Engineering, School of Science and Technology, Nottingham Trent University, Nottingham, NG11 8NS UK; 3 School of Biomedical Sciences, University of Leeds, Leeds, LS2 9JT UK; 4 School of Mechanical Engineering, University of Leeds, Leeds, LS2 9JT UK

**Keywords:** Soft sensors, Soft functional materials, Capacitive sensors, Soft composite polymers, Batch fabrication, High dielectric constant, Engineering, Biomedical engineering, Mechanical engineering

## Abstract

Soft capacitive sensors are essential for emerging technologies in soft robotics, wearable electronics, and human-machine interfaces. However, scalable, customizable, and cost-effective fabrication methods remain a challenge. In this work, we present a novel batch fabrication approach for producing thin, highly sensitive soft capacitive sensors using high-dielectric-constant composite elastomers. Our method enables the rapid production of large-area sensor sheets that can be precisely patterned into arbitrary geometries using laser cutting, eliminating the need for complex microstructuring or lithography. These sensors are composed entirely of soft layers, including carbon nanofiber-based electrodes and dielectric layers enhanced with barium titanate (BTO) or titanium dioxide (TiO_2_). We demonstrate a gauge factor of 2.83 and a sensitivity of 0.55 kPa⁻¹, with low hysteresis and broad pressure range. Furthermore, we present the integration of these sensors into a tactile array for wearable pressure sensing, highlighting their potential in soft robotics and health monitoring systems. This work offers a scalable and versatile platform for next-generation soft sensing technologies.

**Supplementary Information:**

The online version contains supplementary material available at 10.1038/s41598-025-27387-x.

## Introduction

Innovating novel functional materials has catalysed the creation of soft sensors by bringing a range of desired properties^1–8^. These engineered materials enable flexibility, stretchability, and adaptability to arbitrary geometries, while also possessing electrical sensing capabilities. Soft sensors based on such materials can be fully printed using customized, scalable, and rapid processes. Moreover, these materials exhibit biocompatibility due to their non-toxic properties, making them suitable for applications requiring safe human-robot interactions^9–13^. Consequently, soft sensors find diverse applications in fields such as soft robotics^11,14–16^, electronic skin^17–22^, wearable electronics^23–28^ and health monitoring implementable devices^29–32^.

Capacitive sensors are widely utilized for pressure and strain sensing due to their high sensitivity, repeatable response, simple readout electronics, and straightforward design^6,33–35^. There is a growing interest in soft capacitive sensors that rely on completely soft functional materials such as polymeric conductors or conductive liquids embedded in an elastomer^36–45^. These sensors are typically constructed by sandwiching a dielectric material between two soft conductive electrodes. They operate by measuring changes in capacitance resulting from applied force or pressure^46^.

A diverse range of materials has been utilized in the fabrication of soft conducting electrodes. These materials include soft materials with metal coatings or films^47–55^, liquid metals embedded in an elastomer^56–58^, conductive fabrics^59–63^, conductive hydrogels^64–72^ and composite polymers with conductive fillers^73–75^. Metal-coated layers consist of flexible substrates like polyethylene terephthalate (PET) film coated with an indium tin oxide alloy^53,76^ or gold nano-films sputtered onto a polydimethylsiloxane (PDMS) substrate^49^. While these metal layers offer high conductivity and flexibility, they may restrict the stretchability of the sensor. On the other hand, encapsulated liquid metals such as mercury, eutectic gallium indium alloy (EGaIn) and ionic liquids in the elastomeric material combine the conductivity of the metal and the compliance and stretchability of the elastomer to create soft sensor conductors. For example, eutectic gallium indium injected into two intertwined fibres was used to fabricate a capacitive transducer for strain and torsion measurement^57^. However, the application of liquid metals in capacitive sensing for biomedical devices is limited due to factors such as cost, fabrication complexity, and the risks of leakage and toxicity^11^. Recently, conductive fabrics have also been integrated with elastomers to construct soft capacitive sensors. Although these fabrics can conform to complex surfaces in wearable sensors for health monitoring, they may reduce stretchability^61^.

Carbon-based substances and their composites with soft polymers offer highly conductive, reliable, and relatively inexpensive materials for sensor electrodes. These carbonic materials include carbon black^4,74,77–79^, carbon nanofibers (CNF)^80–86^, graphene nanoplatelets^81,87,88^ and graphite^43^. Among these, CNF provides high conductivity and can be easily integrated into elastomeric matrices through shear blending. The resulting composite exhibits excellent conductivity and maintains its conductive properties under loading. The cross-connections of the carbon fibres ensure good conductivity for CNF-filled soft polymers under both tension and compression^89^.

Despite the recent interest in using highly conductive polymers, such as poly(3,4-ethylenedioxythiophene) polystyrene sulfonate (PEDOT: PSS)^1,90–92^, CNF-based capacitors remain a reliable and robust sensing method characterized by high stretchability, sensitivity, and low hysteresis. For instance, Song et al. proposed a PDMS sponge coated by carbon nanotubes (CNT) to serve as an electrode for a capacitor^85^. The capacitive sensor, utilizing a polyvinyl alcohol/phosphoric acid (PVA/H3PO4) gel electrolyte and a separator membrane, exhibited a capacitance change of approximately 25% under a 50% compression strain. Similarly, capacitive sensors utilizing multi-walled carbon nanotubes (MWCNT) have demonstrated high sensitivity, reaching a high sensitivity of 1.33 kPa^− 1^. However, these sensors are constrained by a limited pressure range of 800 Pa^83^.

To constitute the dielectric layer of soft capacitive sensors, various materials have been utilized, including polyethylene, PDMS, nitrile, silicone rubber, and doped elastomers^1,43,81^. Shi et al.. employed a soft Ecoflex layer as a dielectric material sandwiched between PEDOT: PSS electrodes^1^. In another study, a capacitive pressure sensor was fabricated by integrating two mineral hydrogel films composed of very small amorphous calcium carbonate nanoparticles with a polyethylene dielectric film, exhibiting a sensitivity of 0.17 kPa^− 1^, 17% capacitance change under a 1 kPa compression^64^. The sensitivity of soft capacitive sensors can be further enhanced through: (1) the formation of a foam or sponge structure for the dielectric layer, providing higher compressibility and sensitivity^44,48,85,93^, and (2) the incorporation of fillers within the elastomeric dielectric to augment the dielectric constant^90,94–98^.A porous structure of boron nitride/PDMS has been developed to enhance the pressure sensitivity to 0.854 kPa^− 1^ at pressures below 0.5 kPa and to 0.29 kPa^− 1^ for 0.5–2.1 kPa pressure range^44^. However, challenges persist regarding the complex and expensive microstructuring of the dielectric layer^48,93,99–102^. Alternatively, dispersing fillers in the elastomeric dielectric, such as SiO_2_ particles in PDMS provided a rough surface for the dielectric and resulted in higher sensitivity (1.0 kPa^− 1^) compared to PDMS without particles^94^. Nevertheless, the sensor capacitance change remains less than 100% for a compressive strain of 50%.

The fabrication of soft capacitive sensors encompasses a range of techniques, including but not limited to soft lithography methods^103,104^, micro/nanostructuring of layers^48,93,94,99^ and planar coating^47,54,100^. For instance, a soft sensor with a sensitivity exceeding one was proposed by templating PDMS from Calathea zebrine leaves to form a microstructured gel as the dielectric layer^18^. Another capacitive sensor was developed using soft lithography methods, which involve 3D printing of master moulds^103^. However, significant challenges persist in developing methods for rapid, batch, and customized fabrication of reliable capacitive sensors.

We propose a batch fabrication approach for manufacturing thin and highly sensitive capacitive-based soft sensors with customized geometries, high sensitivity, and a broad pressure range. This fabrication method enables rapid, scalable, and reliable construction of capacitive soft sensors with desired geometries and sizes in a simple way. We characterize the mechanical and electrical properties of soft composite polymers that could serve as alternative electrodes for capacitive sensors, including CNF-Ecoflex 00–30 with different mixing weight ratios. Furthermore, we investigate the mechanical response of dielectric materials, including Ecoflex 00–30, Barium Titanate (BaTiO_3_)-Ecoflex 00–30, and TiO_2_-Ecoflex 00–30, under compression. We demonstrate soft capacitive sensors with various dielectric layers, including Ecoflex 00–30, BTO-Ecoflex 00–30 and TiO_2_-Ecoflex 00–30. We chose Ecoflex due to its high stretchability, softness, skin compliance, and lower modulus compared to materials like PDMS, making it ideal for applications involving direct human contact and large deformations.

We also present the rapid fabrication of sensor arrays with the desired geometry. Indeed, we demonstrate a high gauge factor for the capacitive sensors compared to typical silicone sensors, and we show that the sensors exhibit a linear output compared to alternative soft sensors. By utilizing materials with higher dielectric constants, we achieve a high baseline capacitance, ensuring robustness against parasitic capacitance and thereby enhancing the signal-to-noise ratio. While previous studies have explored soft capacitive sensors using various conductive and dielectric materials, our work introduces a scalable and customizable batch fabrication method that enables the production of thin, multilayered sensors with precise geometries using laser cutting. Unlike conventional microstructuring or lithographic techniques, our approach is cost-effective, rapid, and adaptable to diverse form factors. Additionally, we employ high-dielectric composite elastomers (BTO- and TiO₂-enhanced Ecoflex) to significantly improve sensor sensitivity and signal-to-noise ratio. Unlike many prior works, our sensors use uniformly adhered composite layers of the same base elastomer (Ecoflex 00–30) to ensure strong interlayer bonding and reliability. The integration of these sensors into wearable tactile arrays further demonstrates their applicability in soft robotics, prosthetics, and health monitoring, where conformability, durability, and high performance are critical.

## Materials and methods

### Fabrication

**Fig. 1:**
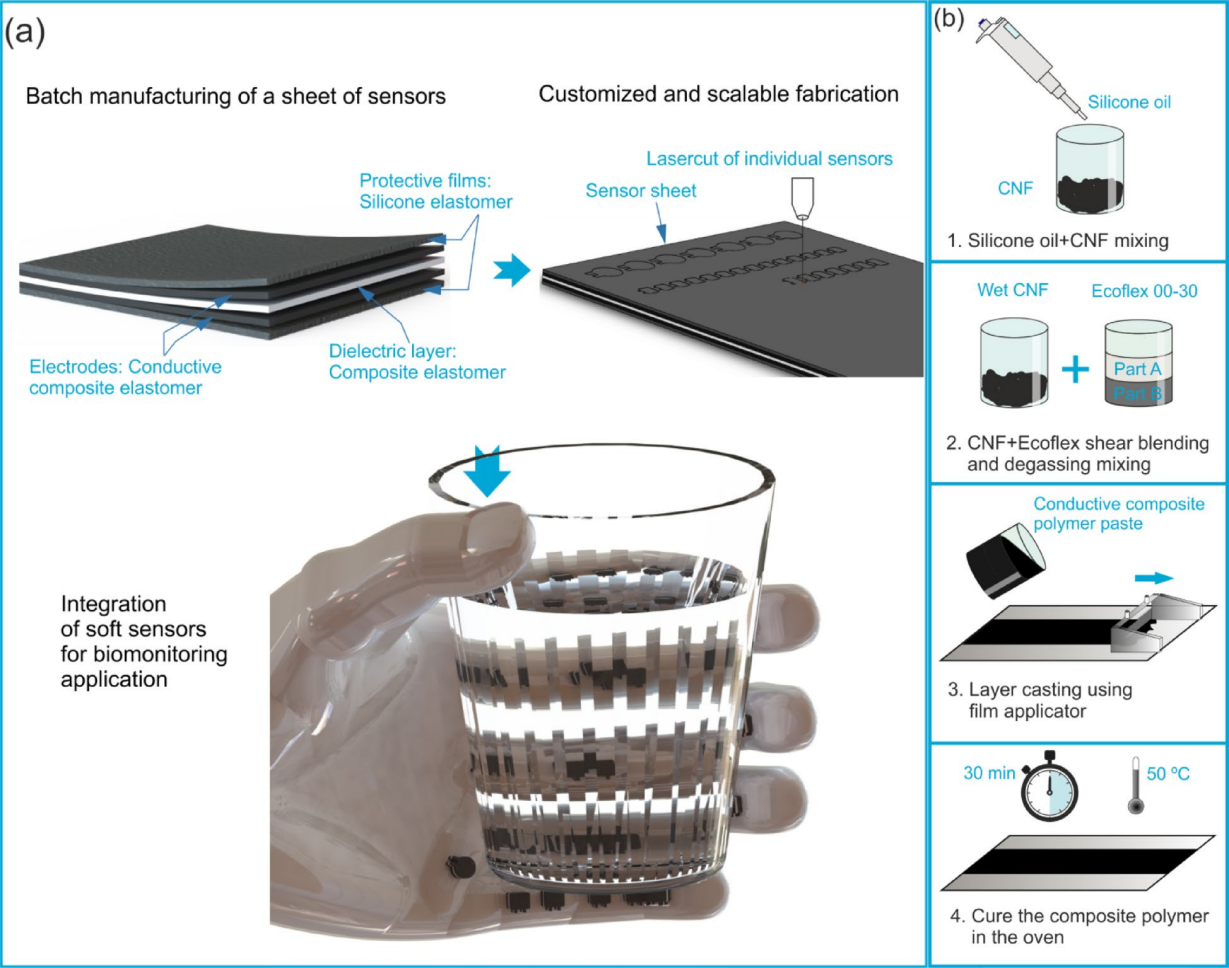
**a** Schematic of the batch and customized fabrication of capacitive sensors using layer-by-layer assembly and laser processing, and their integration onto a hand model. The multi-layer structure consists of protective films, a dielectric layer, and two electrodes, with each layer typically tens of micrometres thick. As all layers are based on the same silicone elastomer composite, they adhere well. **b** Fabrication of the conductive layer: CNF is first blended with silicone oil for dispersion, then mixed with Ecoflex by shear blending, degassed, cast into a thin film, and cured.

The soft capacitive sensor consists of five elastomeric layers, each serving a distinct purpose. Two protective layers are positioned on either side of the sensor to provide electrical insulation for the electrodes, displayed in Fig. 1a. A capacitor is formed by sandwiching the dielectric layer between two conductive layers, and its capacitance changes in response to applied compressive force. This change is directly related to the applied force. Our proposed layer-by-layer manufacturing method enables rapid, reliable, and cost-effective production of large sheets of sensors, scalable for creating thin sensors. Protective, conductive, dielectric, conductive, and protective layers are successively fabricated to construct soft sensors. Each layer is fabricated using a film applicator with a specified gap thickness. Subsequently, the associated laser cutting process offers flexibility in creating sensors with arbitrary geometries and feature sizes as small as a hundred microns. Figure 1a illustrates a potential application of these soft sensors, such as integration onto a human hand for pressure sensing.

The protective layer was fabricated by preparing an elastomeric material through the mixing of parts A and B of Ecoflex 00–30 (Smooth-On, USA) at a weight ratio of 1:1. The resulting slurry was poured onto a flat surface, and a 500 μm thick protective layer was formed by adjusting and applying a film applicator. Subsequently, the coating was cured in the oven at 50 °C for 30 min.

Figure 1b schematically presents the fabrication process of the CNF-silicone elastomer conductive layer. Initially, CNF was mixed with silicone oil using a non-contact planetary speed mixer (ARE-310, Thinky, USA). The addition of silicone oil allows for the integration of CNF into Ecoflex at higher ratios, thereby achieving higher conductivity for the composite polymer. In the next step, the wet CNF was combined with Ecoflex 00–30 at a ratio of 15 wt% through shear blending in the mixer. The resulting paste was degassed and then poured onto the protective layer. The CNF-Ecoflex electrode was formed using a film applicator with the gap adjusted to 500 μm, followed by curing in the oven at 50 °C for 30 min.

**Fig. 2:**
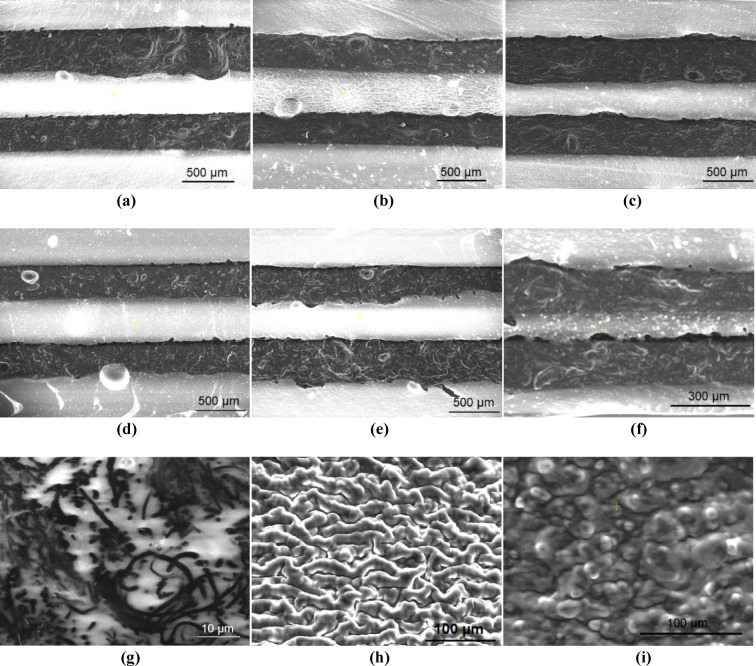
SEM images of fabricated sensors with **a** BTO-Ecoflex 00–30 wt.%12.5, **b** BTO-Ecoflex 00–30 wt.%40, **c** TiO_2_-Ecoflex 00–30 wt.%12.5, **d** TiO_2_-Ecoflex 00–30 wt.%20, **e** Ecoflex 00–30 dielectric with a 500 μm thickness of each layer, and **(f)** BTO-Ecoflex 00–30 wt.%40 200 micron-thick layers. SEM images of **g** CNF-Ecoflex 00–30, **h** BTO-Ecoflex 00–30 wt.%40, and **i** TiO_2_-Ecoflex 00–30 wt.%20 composite layers.

To create the dielectric layer, TiO_2_-Ecoflex 00–30 and BTO-Ecoflex 00–30 composites were prepared by mixing TiO_2_ and BTO nanoparticles with silicone elastomer and degassing the mixture in the mixer. The slurry was then poured onto the conductive layer, spread using the film applicator and cured in the oven at 50 °C for 30 min. The second conductive and protective layers were fabricated in subsequent steps to complete the sensor structure. Figure 2a-f depict SEM images of sensors with 500 μm and 200 μm-thick layers, showing no delamination between layers. This cohesion is attributed to the fact that multiple layers of each sensor are fabricated from composites of the same silicone elastomer, ensuring complete adhesion. Using the proposed method, sensors with layer thicknesses down to 50 μm could be batch-fabricated. However, the viscosity of the composite materials is a limiting factor for fabrication of thinner layers. Figure 2g-i display the CNF-Ecoflex 00–30, BTO-Ecoflex 00–30 wt.%40, and TiO_2_-Ecoflex 00–30 wt.%20, respectively, showing the carbon nanofibers in the elastomeric matrix and the uniform structure of the fabricated composite materials.

### Materials

Bulk sensors were manufactured using a batch fabrication method to ensure rapid, repeatable, and scalable production (Fig. 1). The silicone was prepared by mixing Ecoflex 00–30 (Smooth-On, USA) parts A and B, with 8 g of each component, in a 1:1 weight ratio, producing enough material for a 200 mm × 100 mm × 0.5 mm layer. This mixture was blended at 2000 rpm for 60 s and degassed at 2200 rpm for 90 s using a non-contact planetary mixer (ARE-310, Thinky Mixer, USA). The protective layer was cast from the prepared silicone material using a film applicator set to a gap of 500 μm, followed by curing in an oven at 50 °C for 30 min. For the electrode layer, 2 g CNF was mixed with 9 ml of silicone oil (viscosity 5 cSt, Sigma-Aldrich, USA) at 2000 rpm for 60 s in the mixer. Then, Ecoflex 00–30 parts A and B, with 6.67 g of each component, were added, followed by blending at 2000 rpm for 60 s and degassing at 2200 rpm for 90 s. The resulting paste was cast using the film applicator, with a height adjustment to 1 mm, and cured in the oven at 50 °C for 30 min. The dielectric layer was fabricated using three different materials. The Ecoflex 00–30 dielectric layer was constructed using the same method as described for the protective layer. For TiO_2_-Ecoflex 00–30 wt.%12.5 and wt.%20 dielectric layers, 5 g and 8 g TiO_2_, respectively, were mixed with Ecoflex 00–30 parts A and B, with 20 g of each component. Similarly, for BaTiO_3_-Ecoflex 00–30 wt.%12.5 and wt.%40 dielectric layers, 5 g and 16 g of BaTiO_3_, respectively, were mixed with Ecoflex 00–30 parts A and B, with 20 g of each component. The resulting slurry was poured onto the electrode layer and cast using the film applicator, adjusted to a gap thickness of 1.5 mm, before curing at 50 °C for 30 min in the oven. An additional electrode layer and protective layer were then applied on top of the dielectric layer to create multi-layer capacitive sensors.

The individual sensors were cut from the sensor sheet using a laser cutter (VLS3.50, Universal Laser Systems, USA) to a desired geometry and size (circle of 10 mm diameter with two 2 mm × 3 mm rectangles for connecting wires for sensor characterization) at 40 W power and a 15% speed setting.

### Experimental setup for sensor characterization

A customized testing machine was utilized to evaluate the sensing performance. Individual sensors with a diameter of 10 mm were tested using a cylindrical indenter of matching size. A position-controlled test was conducted to compress the sensor from 0% to 50% strain at a compressive speed of 0.05 mm/s and then uncompressed at the same speed. The capacitance and force of the sensor were measured using FDC1004EVM evaluation board (Texas Instruments, USA) and a Nano25 load cell (ATI, USA), respectively, at a frequency of 100 Hz. The board had four channels required for the characterization of an array of four sensors. We used 60 μm gold wires and silver paste to connect the sensor electrodes to the capacitance measurement device. The protective layer was removed at electrode ends for silver paste connection. The contacts are located outside the pressure-sensitive zone, minimizing interference and parasitic capacitance. This ensures a stable interface during testing.

### Statistical analysis

For material characterization tests, we measured the properties of at least four samples of each material in both compression and tensile tests. The mechanical properties in Table 1 and S1 are presented as mean values $\:\pm\:\:$standard deviation (SD). Error bars on the plots represent the mean values $\:\pm\:$ variance (maximum or minimum variance). For sensors characterization including force and capacitance change analysis, at least four samples of each sensor with different dielectric materials were tested. Sensitivity and gauge factor data were derived from the slope of the regression line. For cyclic tests, at least four samples of each sensor with different dielectric materials were also tested.

## Results and discussions

### Material characterization

Soft capacitive sensors are characterized by their electrical and mechanical properties in determining their sensing performance. The key attributes defining their sensing performance are sensitivity, hysteresis, time response, power consumption and temperature performance^36^. Additionally, the sensor mechanical behaviour is described using force-strain diagrams. The capacitance, $\:C$, of a parallel plate capacitor with an electrode area, *A*, and a dielectric layer thickness, *t*, is given by:

1 $$\:C={\varepsilon}_{r}{\varepsilon}_{0}\frac{A}{t}$$

where $\:{\varepsilon}_{0}$ and $\:{\varepsilon}_{r}$ denote the dielectric constant of vacuum and relative permittivity of the dielectric layer, respectively. We can also obtain the expression for the sensitivity of this capacitor as:

2 $$\:\frac{dC}{dt}=-{\varepsilon\:}_{r}{\varepsilon\:}_{0}\frac{A}{{t}^{2}}$$

**Fig. 3:**
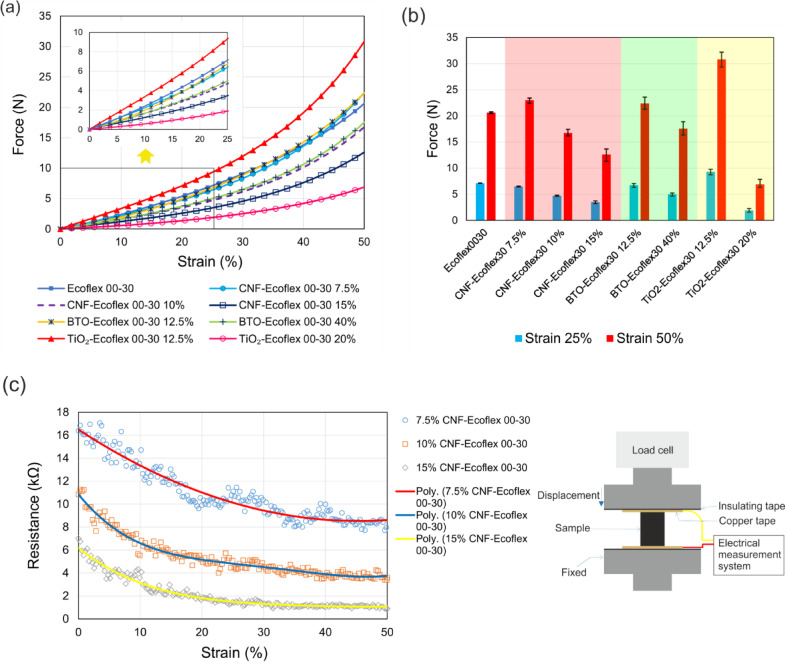
**a** Mechanical characterization of conductive and dielectric composite polymers under compression up to 50%. The inset displays a zoomed-in window of the first 25% strain. The results represent the average value for six samples of each material. **b** The force developed in the samples at 25% and 50% strain, depicted with error bars for various conductive and dielectric materials. **c** Resistance measurement of the conductive CNF-Ecoflex wt.%7.5, 10, and 15 composite polymers. The inset features a schematic of the mechanical and electrical measurement setup.

Equations (1) and (2) indicate that the dielectric material significantly influences both the capacitance and sensitivity of capacitive sensors. On the other hand, the time response of a capacitive sensor is given by $\:\tau\:=RC$, where *R* represents resistance. Lower resistance, achieved through higher conductivity in the capacitive sensor electrodes, results in a shorter time response, which is desirable. Therefore, we first characterize the conductive and dielectric materials used in sensor fabrication. To investigate the mechanical and electrical properties of the conductive material, CNF-Ecoflex 00–30 composites of different weight ratios of %7.5, %10, and %15 were fabricated using the method described in the section “Materials and methods”. Cylindrical samples with a diameter of 17.8 mm and a height of 25 mm were prepared by molding. These samples underwent compression testing using a mechanical testing machine (Instron 5943, Instron, USA) at a speed of 10 mm/min and a strain ranging from 0 to 50%. Compressive force and strain were recorded at a frequency of 50 Hz using a 500 N load cell. Additionally, compression tests were performed to characterize dielectric materials including Ecoflex 00–30, TiO_2_-Ecoflex 00–30 composites with weight ratios of %12.5 and %20, and BTO-Ecoflex 00–30 composites with weight ratios of %12.5 and %40. The ratios were chosen based on the maximum weight ratios that the fillers could be properly dispersed within the elastomeric matrix. The force-strain results are depicted in Fig. 3a for both conductive and dielectric materials. The data presented in the graphs represent the average for six samples for each material. The addition of fillers to Ecoflex 00–30 as the matrix stiffen the material up to a certain percentage, whereas higher ratios of additives soften the composite. For example, the CNF-Ecoflex wt.%15 is roughly twice as soft as the elastomer itself. It is noteworthy that the integration of silicone oil contributes to the softness of the conductive composite. On the other hand, the soft material leads to a large displacement of the dielectric under pressure, resulting in enhanced sensitivity. However, we observed that adding more than 20% TiO_2_ severely compromises the cross-linking between polymer chains, resulting in a compound that is weak and difficult to cure. Additionally, stress-strain data for the compressive tests are shown in Fig. S1 (Supplementary Information). The stress-strain results are summarized in Table 1, providing the compressive modulus and the stress generated in the samples at 20% and 50% strain. The compressive modulus and stress decreases as the CNF ratio increases, from 926.1 Pa at 7.5% to 488.6 Pa at 15%. This indicates that higher CNF concentrations reduce the stiffness of the Ecoflex, making the composite softer and more compliant under compression. Similarly, the stress levels at both 20% and 50% strain decrease with increasing CNF content. For BTO-Ecoflex composites, the compressive modulus initially remains close to that of pure Ecoflex at 12.5% BTO (1022.9 Pa) but decreases significantly at the 40% concentration (716.6 Pa). The BTO-Ecoflex wt.%40 composite also shows a significant decrease in stress at both 20% and 50% strain, implying increased compliance and softening with high BTO content. The TiO_2_-Ecoflex composites demonstrate contrasting behavior to BTO, with the wt.12.5% TiO_2_ ratio showing a significantly higher compressive modulus (1404.0 Pa) than pure Ecoflex, indicating increased stiffness. However, at wt.20% TiO_2_, the modulus sharply decreases to 309.9 Pa, suggesting that beyond a certain concentration, the TiO_2_ particles may disrupt the material matrix, reducing stiffness.

**Table 1:** Mechanical properties of conductive and dielectric composite polymers in compression test.

	Compressive modulus (Pa)	Stress at 20% strain (kPa)	Stress at 50% strain (kPa)
CNF-Ecoflex 00–30 7.5%	926.1	$\:19.47\pm\:0.19$	$\:92.43\pm\:1.41$
CNF-Ecoflex 00–30 10%	693.4	$\:14.15\pm\:0.26$	$\:67.2\pm\:1.98$
CNF-Ecoflex 00–30 15%	488.6	$\:10.43\pm\:0.65$	$\:50.65\pm\:3.87$
Ecoflex 00–30	1029.1	$\:21.60\pm\:0.14$	$\:82.88\pm\:0.41$
BTO-Ecoflex 00–30 12.5%	1022.9	$\:19.74\pm\:1.91$	$\:89.84\pm\:5.17$
BTO-Ecoflex 00–30 40%	716.6	$\:14.92\pm\:0.64$	$\:70.52\pm\:2.62$
TiO_2_-Ecoflex 00–30 12.5%	1404.0	$\:28.48\pm\:0.93$	$\:123.78\pm\:3.95$
TiO_2_-Ecoflex 00–30 20%	309.9	$\:5.44\pm\:0.80$	$\:27.10\pm\:2.24$

Figure 3b presents the mean value of the forces developed in samples under pressure at 25% and 50% strain, along with maximum variance errors. The error is the lowest for Ecoflex 00–30 force: -0.59% and + 0.46% at 25% strain, and − 0.9% and 0.63% at 50% strain. However, integrating fillers into the silicone increases the errors, reflecting variance between samples. For instance, the maximum error for the force at 50% strain becomes 1.79%, 4.05%, and 8.23% for CNF-Ecoflex wt.%7.5%, 10%, and 15%, respectively. The variance in results indicates that achieving uniform dispersion of filler material becomes more challenging as the mixing ratio increases, resulting in materials with fluctuating properties. TiO_2_-Ecoflex wt.%20 composite exhibits the highest variation between materials, with a 13.79% error between samples force at 50% strain.

We evaluated the electrical performance of conductive materials by measuring the resistance change of samples under loading during the compression test. The data were obtained using a Wheatstone bridge circuit connected to the analog input of a data acquisition (DAQ) card (myRIO, National Instruments, USA) setup. We used a copper tape interface to ensure a robust electrical connection to each sample. The tape was isolated from the compression plates and connected to the Wheatstone measurement circuit, as shown in Fig. 3c. Electrical resistance measurements for composites with three different ratios of CNF to Ecoflex are presented in Fig. 3c. The results demonstrate that the resistance of samples decreases with applied pressure, indicating an increase in material conductivity with an increase in compressive strain. Specifically, the conductivity of the CNF-Ecoflex 00–30 wt.%15 composite is higher compared to wt.%10 and wt.%7.5., with resistance starting from ~ 6 kΩ and reducing to ~ 1 kΩ at 50% strain. Hence, we fabricated all the electrodes in sensor characterization using CNF-Ecoflex 00–30 wt.%15 to minimize the power loss. The conductive and dielectric composite polymers were also tested using a tensile test. A dumbbell-shaped sample was used (Fig. S2), and the force–strain and stress–strain results were obtained, as shown in Fig. S3 and summarized in Table S1. The electrical properties of the materials under tensile testing are presented in Fig. S4.

### Sensor characterization

After characterizing the constituent materials, the overall sensor is evaluated to understand the important characteristics of the developed sensors. When the capacitive sensor is compressed to a specified strain, the initial thickness ($\:{t}_{0}$) of a rectangular element of the capacitor changes to $\:(1-\varepsilon\:){t}_{0}$, while the length ($\:{l}_{0}$) increases to $\:\left(1+{\nu\:}_{\mathrm{e}\mathrm{l}\mathrm{e}\mathrm{c}\mathrm{t}\mathrm{r}\mathrm{o}\mathrm{d}\mathrm{e}}\right){l}_{0}$ and the width ($\:{w}_{0}$) decrease to $\:\left(1-{\nu\:}_{\mathrm{d}\mathrm{i}\mathrm{e}\mathrm{l}\mathrm{e}\mathrm{c}\mathrm{t}\mathrm{r}\mathrm{i}\mathrm{c}}\right){w}_{0}$, where $\:{\nu\:}_{\mathrm{e}\mathrm{l}\mathrm{e}\mathrm{c}\mathrm{t}\mathrm{r}\mathrm{o}\mathrm{d}\mathrm{e}}$ and $\:{\nu\:}_{\mathrm{d}\mathrm{i}\mathrm{e}\mathrm{l}\mathrm{e}\mathrm{c}\mathrm{t}\mathrm{r}\mathrm{i}\mathrm{c}}$ are the Poisson’s ratios of the electrode and dielectric layer, respectively. For a soft sensor, the capacitance upon loading would change from $\:C={\varepsilon\:}_{r}{\varepsilon\:}_{0}{l}_{0}{w}_{0}/{t}_{0}$ to:

3 $$\:C={\varepsilon\:}_{r}{\varepsilon\:}_{0}\frac{\left(1+{\nu\:}_{\mathrm{e}\mathrm{l}\mathrm{e}\mathrm{c}\mathrm{t}\mathrm{r}\mathrm{o}\mathrm{d}\mathrm{e}}\right){\left(1-{\nu\:}_{\mathrm{d}\mathrm{i}\mathrm{e}\mathrm{l}\mathrm{e}\mathrm{c}\mathrm{t}\mathrm{r}\mathrm{i}\mathrm{c}}\right)l}_{0}{w}_{0}}{(1-\varepsilon\:){t}_{0}}$$

Equation 3 illustrates how the mechanical properties of the electrode and dielectric materials, along with the permittivity of the dielectric layers, influence the capacitance of the sensor.

**Fig. 4:**
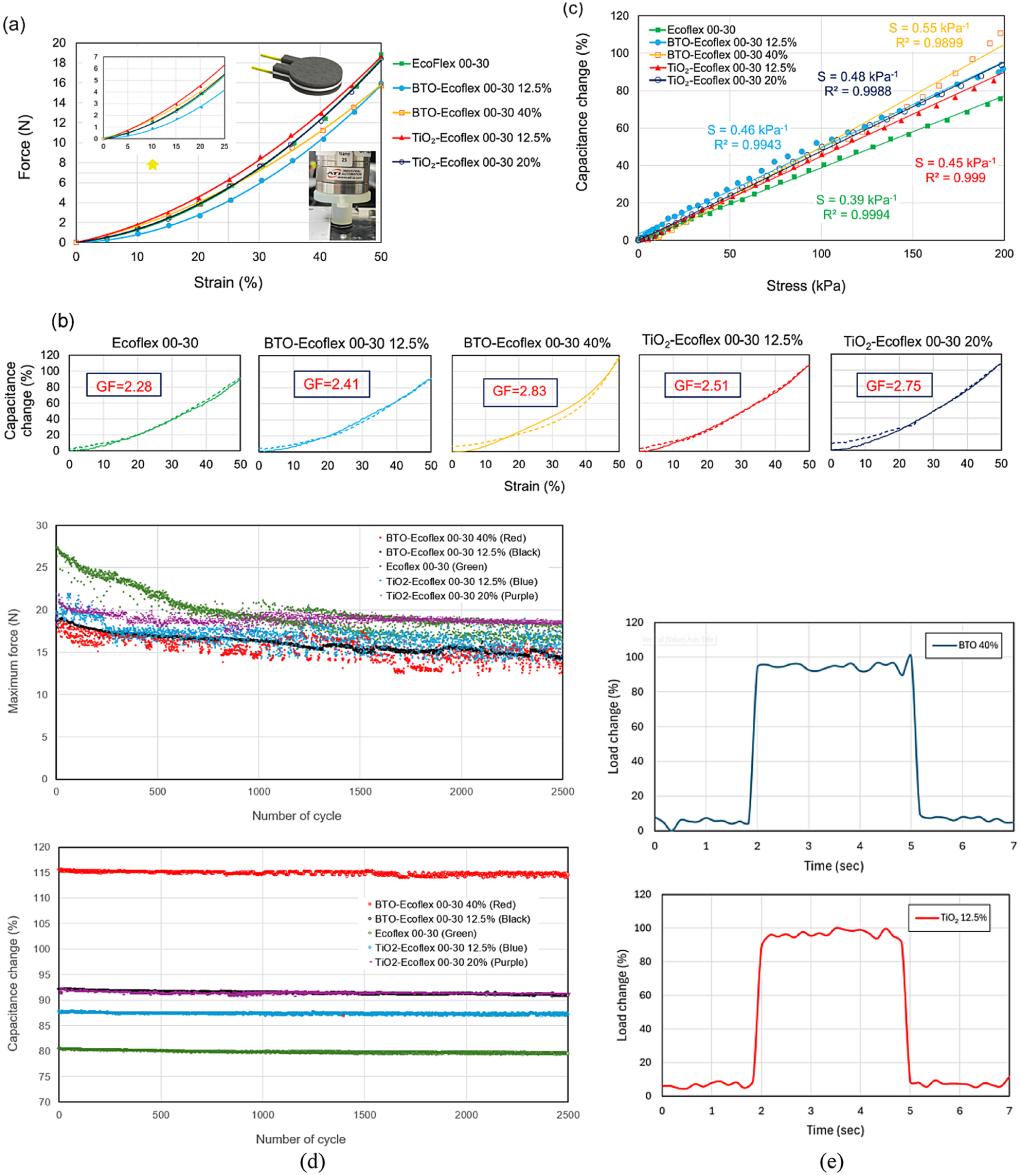
**a** Mechanical characterization of individual sensors under 50% compressive strain. **b** Electrical response of sensors with different dielectric materials. Capacitance change is measured in comparison to the initial capacitance at various strain levels during loading and unloading. The graphs also present data for the related gauge factors. These data have been used to calculate the hysteresis of the sensors. **c** Capacitance change-pressure data of individual sensors under compression from 0% to 50% strain. The sensitivity and root mean square value of a linear fit are displayed in the graph for each sensor. **d** Maximum force and the corresponding capacitance change in sensors with various dielectric materials during 2,500 cycles of cyclic 50% compression strain. **e** Time response graph of sensors with BTO 40% and TiO_2_ 12.5% dielectric layers for obtaining response and recovery times for two example sensors.

Given the high sensitivity and small size of sensors, we developed a customized setup to measure the force, displacement and capacitance synchronously. With this setup, we precisely characterized the mechanical and electrical response of the sensors to compressive forces. An evaluation unit (FDC1004EVM, Texas Instruments, USA) recorded the electrical data, while a customized mechanical tester with a Nano25 load cell (ATI, USA) measured the mechanical properties. Sheets of sensors were prepared using the fabrication method described in Sect. 2, with CNF-Ecoflex 00–30 wt.%15 as the electrode and five different dielectric layers. Then, we laser-cut circular samples with a diameter of 10 mm and two 2 mm × 3 mm rectangular connecting electrodes (Fig. 4a). Each sensor had a total thickness of 2.5 mm, comprising five layers, each 500 μm thick. Gold wires, 60 μm in diameter, were connected to the electrodes using silver paste for capacitance measurement. The samples were loaded using a 10 mm indenter at a speed of 0.05 mm/sec to induce a compressive strain from 0% to 50%, and then unloaded. Figure 4a depicts force and strain measurements for sensor samples with five different dielectric layers: Ecoflex 00–30, BTO-Ecoflex 00–30 wt.%12.5 & 40, and TiO_2_-Ecoflex 00–30 wt% 12.5 & 20. The data indicate that the mechanical properties of the stack of layers closely resemble those of the silicone elastomer.

The unstrained values of capacitance with various dielectric layers of Ecoflex 00–30, BTO-Ecoflex 00–30 12.5%, BTO-Ecoflex 00–30 40%, TiO_2_-Ecoflex 00–30 12.5% and TiO_2_-Ecoflex 00–30 20% were measured at 11.0 ± 0.7 pF, 12.7 ± 0.7 pF, 14.5 ± 1.1 pF, 11.4 ± 1.0 pF, and 12.9 ± 0.7 pF, respectively, based on an average of five samples per variant. Figure 4b illustrates the change in sensor capacitance from the initial state to a 50% strain and in the reverse direction. Sensors with BTO-Ecoflex wt.%40 and Ecoflex 00–30 dielectric exhibit the maximum and minimum capacitance changes at fully strained positions, with values of %116 and %92.4, respectively. The incorporation of BTO with a high dielectric constant into the elastomeric material increases the permittivity of the dielectric layer, thereby enhancing the sensor sensitivity. The data indicate that a 12.5% weight concentration of BTO is insufficient to substantially modify the permittivity of Ecoflex. Conversely, increasing the proportion of TiO_2_ from 12.5% to 20% does not have a significant effect on the sensor sensitivity.

Assuming a linear relationship between capacitance change and strain, we calculated the gauge factor (GF) as $\:\frac{\varDelta\:C/{C}_{0}}{\varepsilon\:}$ from graphs in Fig. 4b. Our sensors achieved a significant gauge factor of 2.83, evaluated between 20% and 50% strains. Figure 4c also depicts data for capacitance change against the applied stress on sensors. Our capacitive sensors exhibit a linear response over a wide range of applied pressure, up to 200 kPa, corresponding to a maximum 50% strain in the sensors. Human body applied pressures span a range from < 10 kPa for a gentle touch to > 100 kPa for body weight, all of which can be covered using our sensors. The sensor with BTO-Ecoflex 40% dielectric shows a high sensitivity of 0.55 kPa^− 1^, as displayed in Fig. 4c. The characteristics of our capacitive sensors have been summarized in Table 2. All sensors exhibit a low hysteresis of less than 10%.

**Table 2:** Performance characteristics of soft capacitive sensors with various dielectric material.

Performance parameter	Dielectric material				
	Ecoflex 00–30	BTO-Ecoflex 00–30 12.5%	BTO-Ecoflex 00–30 40%	TiO_2_-Ecoflex 00–30 12.5%	TiO_2_-Ecoflex 00–30 20%
Gauge factor	2.28	2.41	2.83	2.51	2.75
Sensitivity	0.39 kPa^− 1^	0.46 kPa^− 1^	0.55 kPa^− 1^	0.45 kPa^− 1^	0.48 kPa^− 1^
Hysteresis	4.9%	4.6%	7.0%	5.8%	9.5%

The sensors with different dielectric materials were tested under compressive strain from 0% to 50% and then back to 0% over 2,500 cycles. Figure 4d shows force and strain measurements from the cyclic tests and the corresponding capacitance changes. The results indicate that the sensors exhibit consistent performance in the cyclic tests, with minimal variation over successive cycles.

To determine the response and recovery times, the sensors were compressed from 0 to 1 mm at a high loading speed (15 mm/s). The capacitance changes were recorded, and the response and recovery times were measured to be in the range of 120–167 ms. The results for two sensors, BTO 40% and TiO_2_ 12.5%, are shown in Fig. 4e.

### Sensor arrays

**Fig. 5:**
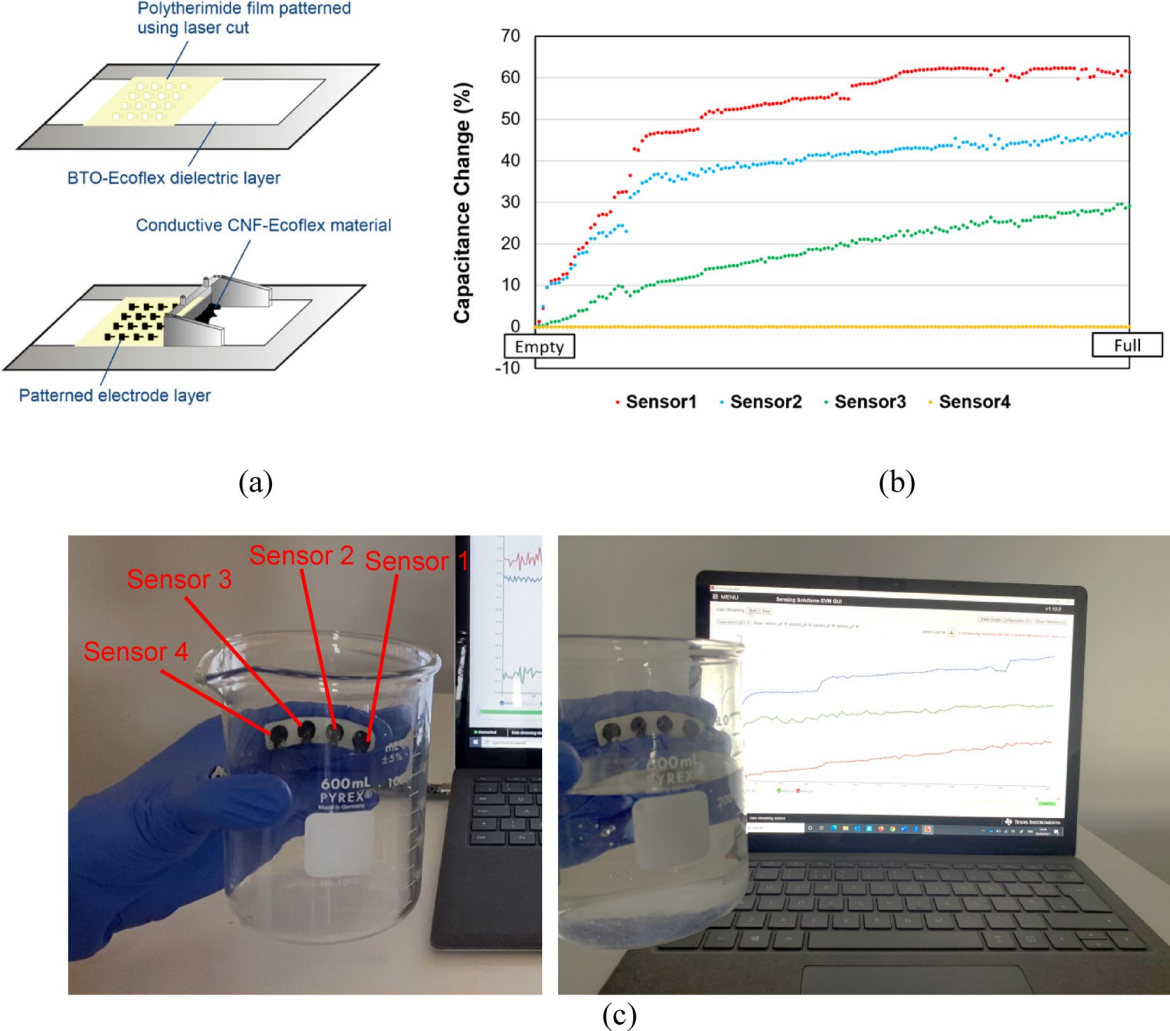
**a** A masking method was used to fabricate the second electrode layer during manufacturing of an array of sensors. The desired size and geometry were laser-cut onto a Polyetherimide film and transferred to the electrode layer by masking the conductive material through the film by applying the applicator. **b** Application of an array of sensors on a hand glove for monitoring pressure in a case study. The subject held a laboratory beaker and then, water was poured into the beaker. Each sensor along the array measured the applied force. **c** Capacitance change of each of four sensors along an array during the pouring of water. The total amount of water poured into the beaker was 350 ml. Sensor 4 did not come into contact with the beaker during the experiment.

Arrays of sensors were fabricated using the material preparation method described in the section “Materials and methods”. We followed the process of developing protective, first electrode, and dielectric layers as previously described. However, for the second electrode layer, we employed a masking technique to create the desired pattern of the sensor array (Fig. 5a). A Polyetherimide film (Goodfellow, USA) with a thickness of 500 μm was applied onto the dielectric layer. Then, the conductive material was prepared and applied using the film applicator through the mask. Subsequently, the masking film was removed, and the patterned electrode layer cured for 30 min in the oven at 50 °C. The proposed method allowed for the achievement of various desired geometries and sizes for the sensors, as we utilized a continuous conductive layer as a ground electrode for all sensors and a patterned electrode layer with a specific design.

We fabricated an array of four sensors, each with a circular shape of 10 mm in diameter and arranged them in a row with a 15 mm distance between each sensor. Based on the higher gauge factor observed for the sensor with the dielectric BTO-Ecoflex 00–30 wt.%40, we constructed the dielectric layer from this composite polymer. To create a tactile sensory system, we adhered the array of sensors to a nitrile hand glove using superglue. A subject was then asked to hold a lab beaker and the sensors’ outputs were recorded while water was poured into the beaker, as depicted in Fig. 5b. The results of capacitance change for the four sensors along the array are shown in Fig. 5c. Sensor number 4, not in contact with the beaker, displays a zero output. By using an array, the force can be monitored along a specific trajectory, allowing for the generation of a pressure profile. This case study demonstrates the application of the sensor array, developed using rapid, reliable, and batch fabrication methods for soft robotics and health monitoring purposes.

## Conclusion

We demonstrated a novel, scalable, and customizable batch fabrication method for soft capacitive sensors, enabling the rapid production of large-area sensor mats that can be precisely patterned using laser cutting. This fabrication approach is distinguished by its simplicity and adaptability: all sensor layers are cast using film applicators and cured in sequence, allowing for consistent layer thicknesses down to tens of microns. The use of a laser cutter for post-processing enables high-resolution customization of sensor geometry without the need for cleanroom facilities or complex lithographic techniques. This makes the method highly suitable for both prototyping and mass production, offering a practical route to scalable manufacturing of soft sensors.

We characterized the electrical and mechanical performance of conductive composite polymers made of carbon nanofibers and silicone elastomer, achieving a low resistance of 6 kΩ for a sample measuring 17.8 × 25 mm, which further reduced to 1 kΩ under 50% compressive strain. Additionally, we developed high-dielectric-constant soft polymers by integrating BTO and TiO₂ into Ecoflex silicone elastomer, resulting in sensors with high sensitivity (0.55 kPa⁻¹) and a large gauge factor (2.83). The sensors exhibited linear response, low hysteresis (4.6–9.5%), and consistent performance under cyclic loading.

In comparison with recent works, we find that sensors using microstructured or porous dielectrics often achieve sensitivities in the range of ~ 0.7–2.8 kPa⁻¹ under low-to-moderate pressures (e.g., ionic hydrogels, MWCNT/PDMS wrinkled microstructures, porous PDMS) with hysteresis values typically between 5 and 7% and response times around 120–167 ms. Our devices show sensitivities of ~ 0.39–0.55 kPa⁻¹ with comparable hysteresis and response/recovery times. Importantly, our capacitive sensors also exhibit relatively high gauge factors (2.3–2.8), exceeding those of many conventional capacitive sensors that typically report GF ≤ 1. This demonstrates that our fabrication method provides competitive sensitivity and stability, and also enhances strain responsiveness without relying on complex microstructuring.

Beyond individual sensors, we demonstrated the fabrication of sensor arrays using a masking technique for patterned electrodes, enabling multi-point tactile sensing. These arrays were successfully integrated into wearable systems, such as gloves, to monitor distributed pressure during object manipulation. This highlights the potential of our sensors in a wide range of applications, including soft robotics, prosthetics, human–machine interfaces, and health monitoring systems. The combination of material performance, fabrication scalability, and application versatility positions this work as a promising platform for next-generation soft sensing technologies.

## Supplementary Information

Below is the link to the electronic supplementary material.

Supplementary Material 1

## Data Availability

Data will be made available upon request from the corresponding authors.
