# Supplementary material for: Customized batch fabrication of highly sensitive thin capacitive soft sensors based on high dielectric constant composite polymers
Source: Sci Rep. 2026 Jan 12;16:1543. doi: 10.1038/s41598-025-27387-x (PMC12800149; doi:10.1038/s41598-025-27387-x)
Supplement: Supplementary file 1 — Supplementary Material 1 [file 41598_2025_27387_MOESM1_ESM.docx]

**Customized Batch Fabrication of Highly Sensitive Thin Capacitive Soft Sensors Based on High Dielectric Constant Composite Polymers**

**A. Ghanbari*, A. A. Colakoglu, A. Serjouei, S. Chakrabarty, R. M. Ichiyama, P. R. Culmer, A. Alazmani***

**Supplementary Material**

We have investigated the mechanical and electrical properties of the conductive and dielectric materials in compression and tensile test. The CNF-Ecoflex 00-30, TiO_2_-Ecoflex 00-30 and BTO-Ecoflex 00-30 composites of different ratios were fabricated using the method described in Section 2 of the paper. The material was then casted into a mould to create a dumbbell sample (shown in Firgure S2) with dimensions as per ISO standard [1]. An Instron machine (Instron 5943, Instron, USA) with a 50 N load cell was used for uniaxial tensile testing at a speed of 100 mm/s at room temperature until the sample ruptured. Figure S3 displays the force-strain results for both conductive and dielectric materials. Table S1 summarizes the mechanical properties of various composite polymers in tensile testing. This includes tensile modulus, tensile strength, and elongation at break for samples made from CNF-Ecoflex 7.5%, 10% and 15%, BTO-Ecoflex 12.5% and TiO_2_-Ecoflex 12.5%.

We also measured the electrical resistance of conductive materials during the tensile test. The electrical resistance was obtained using a Wheatstone bridge circuit connected to the analog input of a data acquisition (DAQ) card (myRIO, National Instruments, USA) setup. A copper tape was used as an interface to ensure a robust electrical connection to each sample and was isolated from the grippers. Figure S4 presents how the electrical resistance of each sample changes with increasing the tensile strain. The results show that the conductivity of both CNF-Ecoflex 00-30 7.5% and 15% decreases with an increase in tensile strain, although the decrease is much sharper in the case of CNF-Ecoflex 00-30 7.5%.


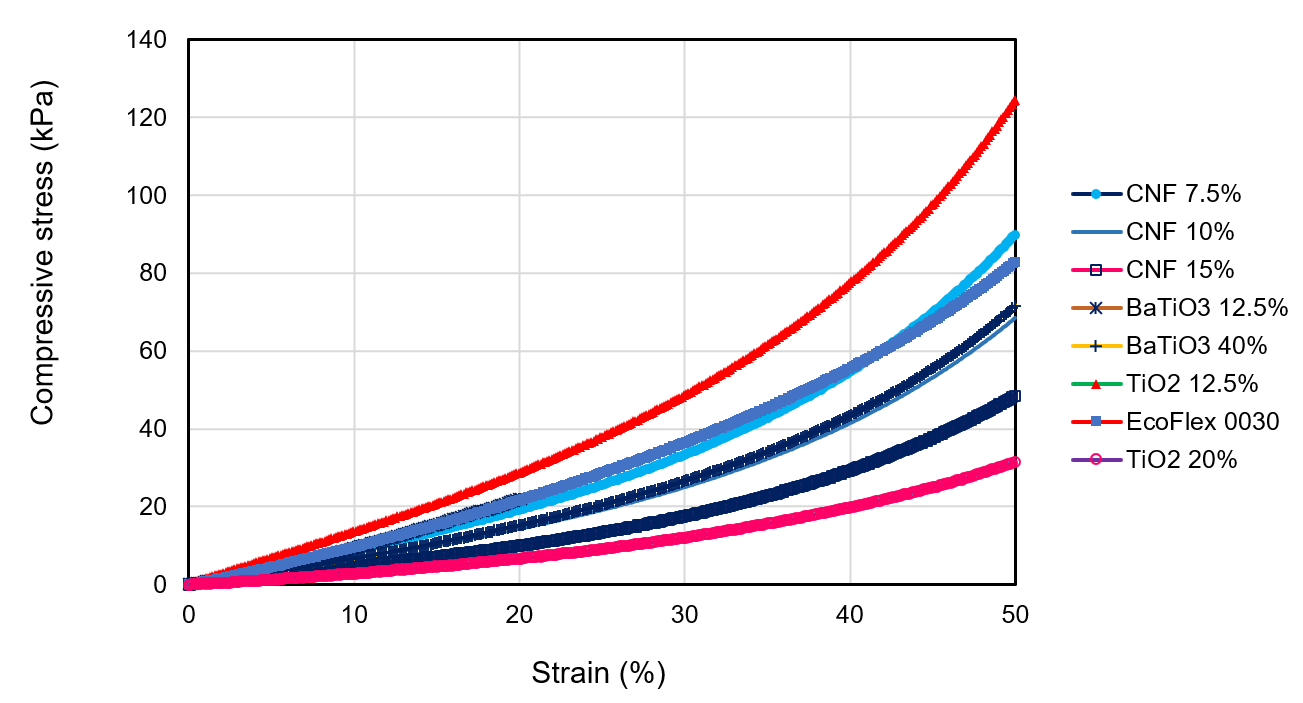


**Figure S1.** Compressive stress developed in conductive and dielectric composite polymers under compression measured in kPa.


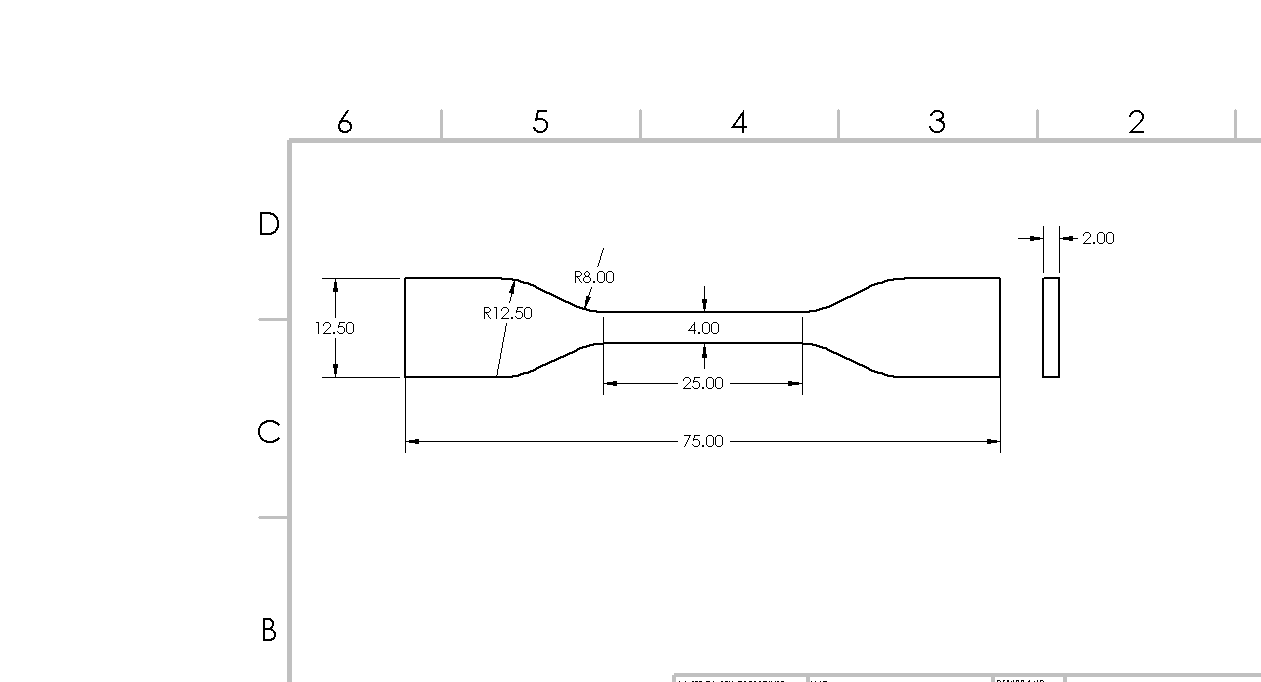


**Figure S2.** Dumbbell sample used for tensile test with dimensions as per ISO standard. All dimensions are in millimetres.


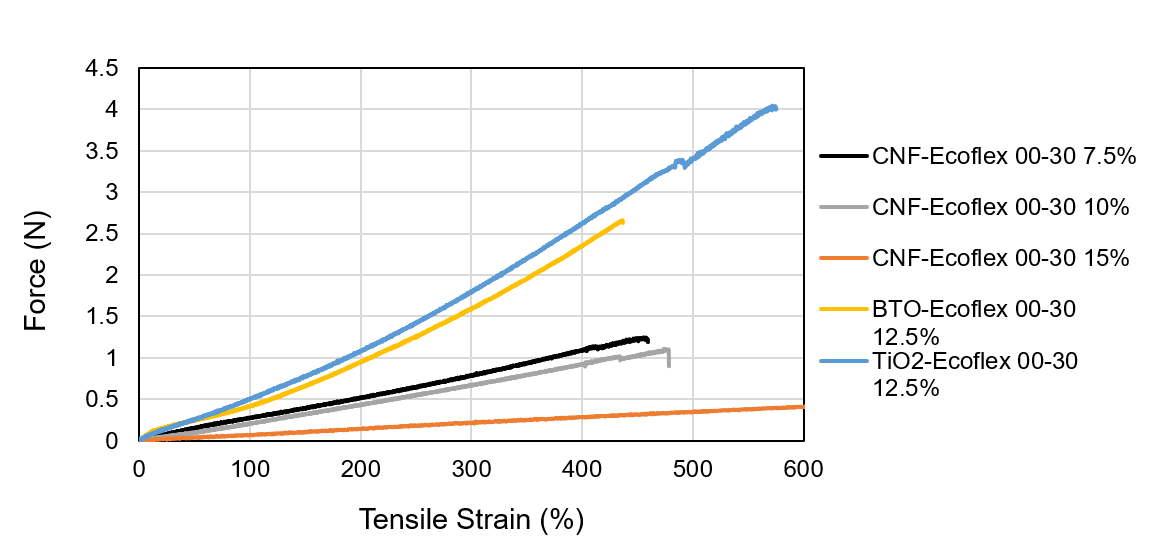


**(a)**


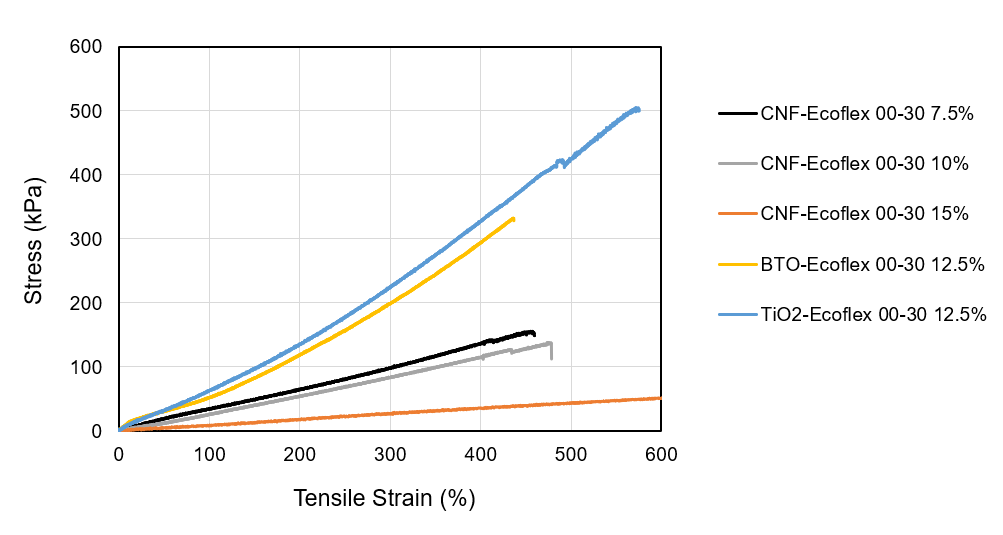


**(b)**

**Figure S3.** Tensile test results of conductive and dielectric composite polymers depicted as: **(a)** axial tensile force, and **(b)** tensile stress, both plotted against tensile strain.

**Table S1.** Mechanical properties of conductive and dielectric composite polymers. Stress values are presented as Mean value $\pm$Standard deviation.

|  | Tensile modulus  (Pa) | Tensile strength (kPa) | Elongation at break (%) |
| --- | --- | --- | --- |
| CNF-Ecoflex 00-30 7.5% | 334.6 | $157.5\pm6.44$ | $459.54\pm22.68$ |
| CNF-Ecoflex 00-30 10% | 282.7 | $139.8\pm1.64$ | $478.46\pm19.55$ |
| CNF-Ecoflex 00-30 15% | 81.6 | $52.8\pm4.74$ | $600.05\pm15.44$ |
| BTO-Ecoflex 00-30 12.5% | 1022.9 | $362.43\pm9.51$ | $436.82\pm4.85$ |
| TiO_2_-Ecoflex 00-30 12.5% | 684.0 | $506.1\pm12.55$ | $575.45\pm3.60$ |


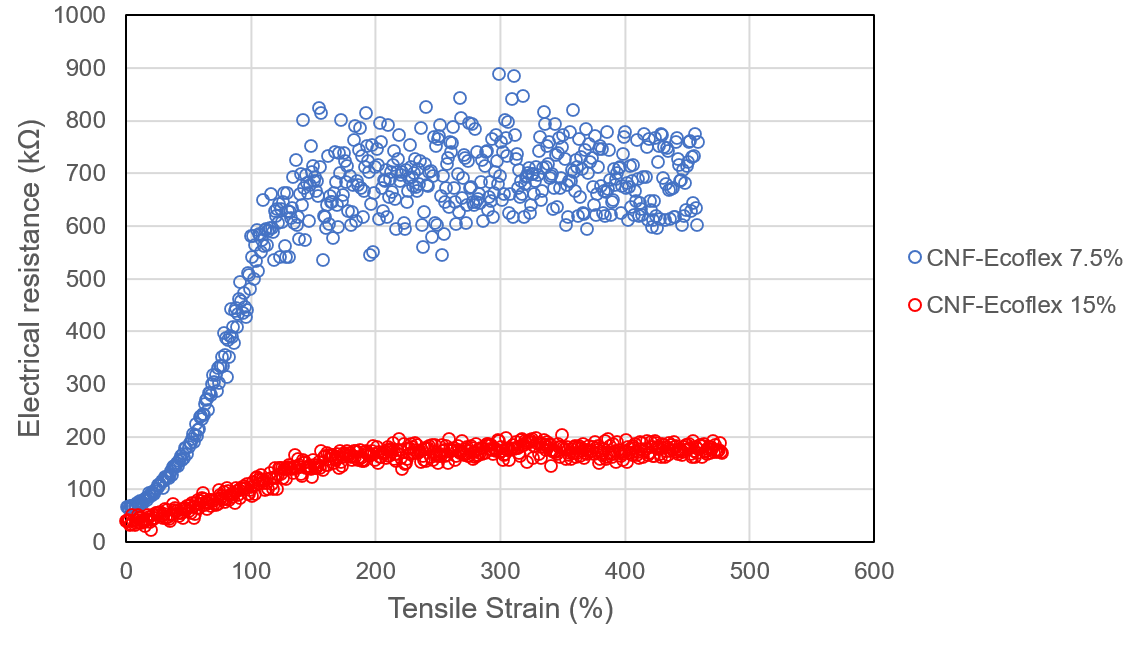


**Figure S4.** Measurement of electrical resistance of samples made of conductive CNF-Ecoflex 00-30 wt.%7.5, and 15 composite polymers in the tensile test.

1. ISO, 37: 2024: Rubber, vulcanized or thermoplastic — Determination of tensile stress-strain properties; ISO: Geneva, Switzerland, 2024.
